# A genome-wide linkage and association study using COGA data

**DOI:** 10.1186/1471-2156-6-S1-S128

**Published:** 2005-12-30

**Authors:** Xiaofeng Zhu, Richard Cooper, Donghui Kan, Guichan Cao, Xiaodong Wu

**Affiliations:** 1 Department of Preventive Medicine and Epidemiology, Loyola University Medical Center, Maywood, IL 60153

## Abstract

**Background:**

Genome-wide association will soon be available to use as an adjunct to traditional linkage analysis. We studied alcoholism in 119 families collected by the Collaborative Study on the Genetics of Alcoholism and made available in Genetic Analysis Workshop 14, using genome-wide linkage and association analyses.

**Methods:**

Genome-wide linkage analysis was first performed using microsatellite markers and a region with the strongest linkage evidence was further analyzed using single-nucleotide polymorphisms (SNPs). Family based genome-wide association test was also conducted using the SNPs.

**Results:**

Nonparametric linkage analysis revealed weak linkage evidence on chromosome 7, and association analysis identified SNP tsc0515272 on chromosome 3 as significantly associated with alcoholism.

**Conclusion:**

Linkage analysis may require large sample sizes and high quality genotyping and marker maps to adequately improve power, while association analysis could hold more promise in efforts to identify variants responsible for complex traits.

## Background

Alcoholism is a complex trait affected jointly by genetic components and environmental factors. Linkage and association are often used to search for the responsible genetic variants of a complex trait. It is believed that association analysis has more power than linkage analysis in the genetic dissection of complex traits such as alcoholism, providing that strong linkage disequilibrium is present between a testing marker and the disease locus [1]. Because of rapid technical improvements and decreasing experimental costs, genome-wide association analysis will soon become as routine as the traditional genome-wide linkage analysis for researchers. To compare the two methods, we performed both genome-wide linkage and association analysis of the Collaborative Study on the Genetics of Alcoholism (COGA) data made available to Genetic Analysis Workshop 14 (GAW14) participants.

## Methods

The COGA dataset included 1,294 White individuals in 119 families. These individuals were enrolled for a linkage and association study. We selected ALDX1 as the phenotype. ALDX1 has five categories: 0: no information; 1: pure unaffected; 2: never drank; 3: unaffected with some symptoms; 5: affected. Fourteen individuals are classified in group 2 (never drank). In our analysis, we then defined 5 as affected, 1 and 2 as unaffected, and the remaining as unknown. The analysis results of coding 2 as unknown were essentially the same as that of coding 2 as unaffected. Our data then consisted of 528 affected individuals, among them, 487 offspring. The data also included 315 microsatellite markers evenly spaced across the genome with average marker distance of about 10 cM. There are also 10,081 single-nucleotide polymorphisms (SNP) across genome genotyped using GeneChip Mapping 10 K Array marker set of Affymetrix Inc.

### Statistical analysis

Both single- and multipoint genome-wide nonparametric linkage (NPL) analyses were performed and the S_ALL _statistic [2] was used to assess the linkage evidence, as recommended by Sengul et al. [3]. We used the microsatellite markers for this genome-wide linkage analysis, with the application of the computer program ALLEGRO, which calculated Kong and Cox's LOD scores [4]. We then performed linkage analysis using SNPs in the region with the strongest linkage evidence to explore whether dense SNP markers could further improve linkage evidence. Three families were split to reduce the computation intensity in the linkage analysis.

We next performed family-based association testing (FBAT) by applying the program FBAT using the SNP [5]. The method implemented in FBAT can test association as well as linkage while avoiding spurious associations caused by population stratification. Because FBAT divides a large pedigree into small nuclear families and multiple sibs in a family are used, we then computed the test statistic using the empirical variance, as described in Lake et al. [6], to protect against type I error.

## Results

We first performed single-point NPL analysis [2] using S_ALL _statistic suggested by Sengul et al. [3]. The LOD scores were converted from NPL Z scores by the method of Kong and Cox [4]. Table 1 summarizes the markers with observed LOD scores ≥ 1.0. The strongest single-point LOD score occurred at marker D7S820 (LOD score 2.6, asymptotic *p *= 0.00027). We also observed five additional markers on chromosome 7 with LOD scores ≥ 1.0. The linkage information for a single marker was lower than multiple markers. We then conducted multipoint linkage analysis and the results were generally consistent with the single-point analyses (Table 1). The largest multipoint LOD score was on marker D7S1870 (LOD score 1.77, asymptotic *p *= 0.002), 13 cM away from marker D7S820. Although the linkage information was improved in multipoint analysis, the observed LOD scores were sometimes lower than the single-point analyses. This is perhaps due to the fact that multipoint linkage analysis is sensitive to genotyping errors and map misspecification [7]. In contrast, single-point analysis is robust to genotyping errors and no marker map information is required, but it is less efficient and more subject to random noise [7]. This can be observed from further linkage analysis using SNP in the region between marker D7S1870 and D7S1817 on chromosome 7, where 188 SNP were genotyped in an interval of 40 cM. For example, we observed 7 SNPs with LOD scores ≥ 1.5 and the largest LOD score 4.07 occurred at SNP tsc0039708 (at 113.922 cM) in single-point analysis. Further analysis revealed that 64% of families did not have information for linkage analysis at the location of SNP tsc0039708, which could explain the large LOD score observed at this SNP [7]. The heterozygosity of this SNP is 0.185. Multipoint analysis resulted in the largest LOD score (2.12 at 101 cM) and was consistent with that using microsatellite markers. The average linkage information using 188 SNPs was increased to 95%. The number of SNPs could apparently be reduced. For example, by selecting the most informative SNP every 0.5 cM, we observed the largest LOD score 1.76 at 110 cM with essentially no loss of linkage information (92%).

We then performed genome-wide association using FBAT on the SNP data. The *p*-values of the test statistic on each SNP were calculated based on the empirical variance, as described in Lake et al. [6]. The procedure can protect against type I error due to FBAT dividing large pedigrees into small nuclear families and using multiple sibs within a family. There were total of 10,081 SNPs across the genome; 417 SNPs were not polymorphic and 423 SNP showed evidence of departure from Hardy-Weinberg equilibrium (*p *< 0.01). These SNPs were excluded from further analyses. We observed 670, 167, and 19 SNPs with *p*-value less than 0.05, 0.01, 0.001, significantly exceeding 457, 91, and 9 SNPs expected under the null hypothesis of no association or linkage, suggesting true association and linkage between SNP and alcoholism. Table 2 presents the 19 SNPs with nominal *p*-values less than 0.001. Interestingly, only two associated SNPs (tsc0668988 and tsc1177811 on chromosome 1) were close to the region where weak linkage evidence was observed. SNP tsc0515272 on chromosome 3 showed the most significant association and linkage evidence to alcoholism (nominal *p*-value = 0.000006) and was close to genome-wide significance (Bonferroni corrected *p*-value = 0.055). For the further comparison with the linkage result on chromosome 7, we also listed the 5 SNPs with nominal *p*-values less than 0.01 in the association analysis. All the 5 SNPs were located at least 28 cM away from the linkage peak.

## Discussion

We conducted genome-wide linkage and association analyses using microsatellite markers and SNPs on the data provided by GAW14. Both single- and multipoint NPL analyses showed suggested linkage evidence on chromosome 7. We could not replicate the linkage evidence on chromosome 3 (LOD = 0.37 at 71 cM) that was reported by Foroud et al. [8]. However our linkage analysis failed to identify a genome-wide significant region linked to alcoholism when using microsatellite markers. Using dense SNP markers will improve linkage information, and theoretically will improve the power to detect linkage. However, it may bring additional challenges compared with using microsatellite markers because high quality genotyping and SNP maps are required and much more computation power is needed. A recent study also suggested that the presence of linkage disequilibrium between tightly linked makers can inflate type I error because the current analysis methods assume linkage equilibrium [9]. Thus, further analysis tools allowing linkage disequilibrium between tightly linked markers need to be developed.

In contrast, association analysis may hold great promise in the genetic dissection of complex traits. In this study, we observed genome-wide significant evidence of SNP tsc0515272 associated with alcoholism after Bonferroni correction for multiple comparisons. Such a correction is usually conservative because of existence of linkage disequilibrium between SNPs located close to one another. Interestingly, we did not observed consistent results between linkage and association analyses. For example, we did not observed significant association evidence for SNPs under the linkage peak on chromosome 7. A possible reason is that the SNP genotypes in this study are still not able to capture all of the genetic variation in this region. Theoretical studies suggest that 250,000–800,000 SNPs are required for a genome-wide association study [10,11]. Some haplotype analysis may improve the current results. Linkage analysis did not reveal significant linkage evidence around SNP tsc0515272, where significant association was found, suggesting the lack of power of the linkage analysis. It should also be caution that type I error from both linkage and association analyses can also contribute the inconsistence of the two methods. We believe that the evidence identified in linkage or association analyses could be the important genetic finding and should be further studied.

## Abbreviations

COGA: Collaborative Study on the Genetics of Alcoholism

FBAT: Family-based association test

GAW14: Genetic Analysis Workshop 14

NPL: Nonparametric linkage

SNP: Single-nucleotide polymorphism

## Figures and Tables

**Table 1:** Peak of single- and multipoint LOD scores observed in the nonparametric linkage analysis using microsatellites

			Single		Multipoint	
				
Chr	Marker	Location (cM)	LOD	Information	LOD	Information
1	D1S226	114	1.17	0.5738	0.44	0.8938
2	D2S1329	4.9	1.04	0.4021	1.26	0.8114
7	D7S673	30.1	1.30	0.6108	1.19	0.9189
7	D7S2846	56.8	1.44	0.5697	1.02	0.8573
7	D7S478	68.9	1.37	0.5314	0.31	0.9117
7	D7S1870	94.2	1.54	0.7205	1.77	0.8982
7	D7S1797	101.9	0.26	0.4912	1.26	0.8809
7	D7S820	107.5	2.60	0.5884	1.05	0.8816
7	D7S821	116.6	0.72	0.5714	1.25	0.8722
7	D7S1796	120.	1.05	0.5079	1.13	0.8742
7	D7S1799	127.7	0.55	0.5300	1.46	0.8451
12	D12S1045	169.8	1.33	0.5739	1.21	0.7670
12	D12S392	177.3	1.35	0.3944	1.44	0.6428
21	D21S1440	36.	0.80	0.4882	1.04	0.6118
21	D21S1446	62.7	1.96	0.5759	1.71	0.6154

**Table 2:** The most significant SNPs identified by FBAT. Empirical variance was used to estimate the *p*-value.

Chrom	SNP	position (cM)	freq	*p *value of (HWE)	No. of fam	S^a^	E(S)^b^	Var(S)^c^	Z^d^	*p*-value^e^
1	tsc0616094	68.9	0.868	1.0	41	221	202.4	26.6	3.6	0.000305
1	tsc0668988	99.4887	0.37	0.39	55	121	146.7	55.2	-3.5	0.000552
1	tsc1177811	105.535	0.318	0.13	53	83	105.4	43.5	-3.4	0.000682
**3**^**f**^	**tsc0515272**	**164.236**	**0.876**	**0.02**	**45**	**248**	**223.4**	**29.6**	**4.5**	**0.000006**
4	tsc0571248	172.977	0.223	0.26	54	79	102.2	49.6	-3.3	0.000988
5	tsc0158479	95.668	0.063	0.50	35	27	43.9	20.6	-3.7	0.000198
6	tsc1175206	127.576	0.675	0.59	59	279	252.9	52.6	3.6	0.000327
6	tsc0608218	146.124	0.184	0.61	48	63	81.7	31.4	-3.3	0.000839
9	tsc0048697	13.7398	0.342	0.87	54	113	135.5	46.2	-3.3	0.000935
11	tsc0569292	6.78451	0.788	0.02	40	191	173	27.3	3.5	0.00055
11	tsc0919042	81.1855	0.15	0.75	49	65	88.5	44.7	-3.5	0.000444
13	tsc0271621	60.1748	0.173	0.59	46	55	81.3	59.5	-3.4	0.000659
13	tsc0056748	73.9934	0.191	1.0	42	28	49.4	28.1	-4.0	0.000053
14	tsc0902508	81.2799	0.14	0.39	40	46	64.9	30.7	-3.4	0.000639
15	tsc0058074	49.9916	0.556	0.89	75	300	331.9	86.3	-3.4	0.000599
16	tsc1750530	59.8297	0.107	0.02	46	44	69.7	45.8	-3.8	0.000147
19	tsc0061923	66.8126	0.885	1.0	26	145	161.1	21.6	-3.5	0.000512
19	tsc0598556	102.054	0.664	0.26	63	335	304.3	73.9	3.6	0.000347
20	tsc0060446	35.4473	0.2	1.0	63	90	118.4	43.2	-4.3	0.000015
7	tsc0331830^g^	33.9373	0.113	1.0	39	59	74.2	31.5	-2.7	0.0067
7	tsc0593964^g^	42.6174	0.629	0.53	70	332	305.8	79.2	2.9	0.00328
7	tsc0042959^g^	44.4931	0.136	0.47	49	57	77.9	50.7	-2.9	0.003313
7	tsc0051325^g^	44.5631	0.749	0.58	61	284	309.8	79.5	-2.8	0.003754
7	tsc0797235^g^	122.213	0.897	0.71	37	231	214.6	37.6	2.7	0.007624

^a ^Observed FBAT statistic based on a linear combination of offspring genotypes and traits.

^b ^Estimated mean of S.

^c ^Estimated variance of S.

^d ^Z score defined by (*S - E*(*S*))/.

^e ^No multiple comparison was corrected.

^f ^Bold text indicates that the SNP achieves genome-wide significance.

^g ^These SNPs listed because of linkage evidence observed on chromosome 7.

## References

[B1] Risch N, Merikangas K (1996). The future of genetic studies of complex human diseases. Science.

[B2] Whittemore AS, Halpern J (1994). A class of tests for linkage using affected pedigree members. Biometrics.

[B3] Sengul H, Weeks DE, Feingold E (2001). A survey of affected-sibship statistics for nonparametric linkage analysis. Am J Hum Genet.

[B4] Kong A, Cox NJ (1997). Allele-sharing models: LOD scores and accurate linkage tests. Am J Hum Genet.

[B5] Horvath S, Xu X, Laird NM (2001). The family based association test method: strategies for studying general genotype-phenotype associations. Eur J Hum Genet.

[B6] Lake S, Blacker, Laird N (2001). Family based tests in the presence of association. Am J Hum Genet.

[B7] Sullivan PF, Neale BM, Neale MC, van den Oord E, Kendler KS (2003). Multipoint and single point non-parametric linkage analysis with imperfect data. Am J Med Genet.

[B8] Foroud T, Edenberg HJ, Goate A, Rice J, Flury L, Koller DL, Bierut LJ, Conneally PM, Nurnberger JI, Bucholz KK, Li TK, Hesselbrock V, Crowe R, Schuckit M, Porjesz B, Begleiter H, Reich T (2000). Alcoholism susceptibility loci: confirmation studies in a replicate sample and further mapping. Alcohol Clin Exp Res.

[B9] Huang Q, Shete S, Amos CI (2004). Ignoring linkage disequilibrium among tightly linked markers induces false-positive evidence of linkage for affected sib pair analysis. Am J Hum Genet.

[B10] Carlson CS, Eberle MA, Rieder MJ, Yi Q, Kruglyak L, Nickerson DA (2004). Selecting a maximally informative set of single-nucleotide polymorphisms for association analyses using linkage disequilibrium. Am J Hum Genet.

[B11] Gabriel SB, Schaffner SF, Nguyen H, Moore JM, Roy J, Blumenstiel B, Higgins J, DeFelice M, Lochner A, Faggart M, Liu-Cordero SN, Rotimi C, Adeyemo A, Cooper R, Ward R, Lander ES, Daly MJ, Altshuler D (2002). The structure of haplotype blocks in the human genome. Science.

